# COVID-19 Pandemic Impact on Confidence to Return to Sport: Perspectives From National Collegiate Athletic Association (NCAA) Athletes

**DOI:** 10.7759/cureus.39824

**Published:** 2023-06-01

**Authors:** Katia E Valdez, Ashvita Ramesh, Michael A Terry, Vehniah K Tjong

**Affiliations:** 1 Department of Orthopaedic Surgery, Northwestern University Feinberg School of Medicine, Chicago, USA

**Keywords:** ncaa, division one collegiate athlete, covid-19, return to play, return to sport

## Abstract

Background: One of the unforeseen impacts of the COVID-19 pandemic has been a decrease in athletes’ confidence to return to their sport after mandates were lifted. Both physical and psychological effects have been implicated. This study aimed to measure the severity of these changes among a group of National Collegiate Athletic Association (NCAA) athletes.

Methods: A novel *Readiness to Return to Sport Survey, *based on the validated ACL-RSI survey, was distributed to Division 1 collegiate athletes. The survey evaluated the psychological readiness of each player to return to sport in the context of the COVID-19 pandemic, utilizing a 1-10 scale (1 = least confident and 10 = most confident). Numerical responses to each survey were summed to create a primary outcome score-an athlete’s *Return to Sport Readiness Score*. Higher scores indicate higher levels of readiness to return to sport in the nearest coming season.

Results: Responses came from 68 athletes representing a variety of sports. Of those with an injury, 14 (82.35%) attributed their injury to changes in their training schedule due to COVID-19 restrictions, and the remaining three (17.65%) did not. Among all athletes, the mean return to sport readiness (RTS) score was 44 (SD 24.76). Those playing a winter sport had the lowest mean RTS score, 35 ± 23, and those playing a fall season sport had the highest mean score, 48 ± 25.97. Overall, competitive athletes on leave from the sport due to collegiate and Division 1 COVID-19 guidelines had lower reported mean RTS scores as compared to athletes outlined in many other anterior cruciate ligament return to sport after injury survey (ACL-RSI) studies.

Conclusions: Overall, the athletes surveyed in our study reported much lower levels of readiness to return to sport in the context of COVID-19 than athletes surveyed in other studies, exhibiting COVID-19’s unique impact on their confidence to return to their scheduled sport season. These differences may highlight the COVID-19 pandemic as a more severe detriment to returning to sport readiness among division-one athletes than recovering from injury alone. Given such an impact, more research is needed to elucidate the percentage of these athletes that returned to or abstained from their sport, as well as any motivating, facilitating, or detrimental factors in their choice.

## Introduction

The coronavirus disease 2019 (COVID-19) pandemic has undoubtedly disrupted daily life on a global scale. As universities were forced to close their doors, many athletes’ training programs were reworked or temporarily halted to abide by the Centers for Disease Control (CDC) and university guidelines. During the height of the stay-at-home order in 2020, numerous sporting competitions were postponed and canceled due to the risk of viral spread among athletes and spectators. During this time, many athletes limited their training time to under 60 minutes and 80% of their maximum ability, as prolonged and strenuous training has been shown to weaken the immune system and increase the risk of infection [1]. These closures resulted in reduced training and increased time off for athletes, potentially causing reductions in physical fitness, muscle mass, and athletic performance [2].

The long-term cardiovascular consequences of COVID-19 are also unclear, and the question of eligibility for return to sport following asymptomatic or symptomatic COVID-19, with or without suspected myocardium involvement, is critical [3]. Of note, the impact of the COVID-19 pandemic is especially pervasive regarding injured athletes’ confidence to return to sports post-injury recovery. A 2019 study found that injured athletes who experience significant pre-injury adversity are often overwhelmed by their injury and lack healthy coping mechanisms [4]. This suggests that both the psychological burden of the COVID-19 pandemic and its physical impact on training may thwart athletes’ abilities to recover from their injuries. As a result, impacted athletes may be left with diminished confidence in their ability to return to sport (RTS) at their pre-injury capacity [4]. It has been demonstrated that an athlete’s psychological response to injury and recovery impacts their return to play and the length of their recovery [5]. In the context of COVID-19 exposure, athletes are removed from their support networks and normal training schedules [1]. For many, these routines are essential for mental health hygiene, helping some manage their depression or anxiety [1]. With high economic and competitive pressure to return to sports post-injury [6], it is no surprise that athletes reported more psychosocial than functional or physical barriers to returning to their sport before COVID-19 [7]. With the implementation of stay-at-home orders during the pandemic, these psychosocial barriers to returning to sports were likely exacerbated.

Returning to sport before an athlete is emotionally prepared may also worsen psychological symptoms; this underscores the importance of mental readiness and fear avoidance questionnaires in assessing an athlete’s comfort and confidence in returning to play [8]. Perhaps the most widely distributed of such questionnaires is the injury-psychological readiness to return to sport (I-PRRS), which assesses confidence to return to sport after injury by asking athletes to rate six items: confidence to play, confidence to play without pain, confidence to give 100% effort, confidence to not concentrate on prior injury, confidence in the capability of the injured body part, and confidence in skill level/ability. These are rated on a scale from 0 (no confidence) to 100 (complete confidence) [9]. Another measure, the Athlete Fear Avoidance Questionnaire (AFAQ), measures injury-related fear avoidance and can be used to identify psychological barriers to returning to sport [10]. Additional methods, including the Tampa Scale of Kinesiophobia (TSK) [11] and the anterior cruciate ligament return to sports after injury (ACL-RSI) scale [12], show additional promise in determining readiness to return to sport.

This study aims to understand how the pandemic affected athletes by using an augmented version of the ACL-RSI for its high validity in the context of COVID-19. We hypothesize that the pandemic has considerably impacted athletes’ fitness, injury recovery, and confidence in their ability to return to and perform their sport.

## Materials and methods

Study design

This was a cross-sectional survey of Division I collegiate athletes in Chicago, IL. Participants were eligible if they were 18 years of age or older, appeared on their collegiate team’s player roster, anticipated returning to their sports in the 2020-2021 Fall, Winter, or Spring athletic seasons, and were willing and able to provide informed consent. Fall sports include Field Hockey, Soccer, Football, Cross Country, Basketball, and Volleyball. Winter sports include Diving, Swimming, Golf, and Fencing. Spring sports include Baseball, Lacrosse, Track, and Softball. First-year students and those not meeting inclusion criteria were excluded from the study. Eligible participants were provided a REDcap v.10 survey link for the ACL-RSI augmented survey to complete online at their leisure.

Study outcomes and covariates

A readiness to return to sport survey (RTS survey) was distributed, which evaluated the psychological readiness of each player to return to sports in the context of the COVID-19 pandemic and assessed COVID-19’s impact on the player. Our RTS survey (Appendix A) is based on the ACL-RSI, an assessment tool originally designed and validated in 2008 to assess athletes’ readiness to return to their sport after ACL repair surgery. The original target population of the ACL-RSI survey was athletes taking a leave from their sport due to surgery. We chose to modify this tool for athletes abstaining from their sport due to COVID-19 pandemic closures and Division I safety regulations. Some of the athletes we administered this survey to were also recovering from injuries that kept them from training and competing as they had before.

The novel RTS survey utilizes a 1-10 scale (1 = least confident and 10 = most confident). The numerical responses to each survey were summed individually to create a primary outcome score-an athlete’s return to sport readiness score. Overall, the highest possible RTS score was 100 (maximum score of 10 per question, 10 total questions), and the lowest possible score was 0 (minimum score of 0 per question, 10 total questions). Higher scores indicate higher levels of readiness to return to sport in the nearest coming season. Additional patient demographic data, including age, specified gender, race, and ethnicity, were collected. The additional variables of type of sport played, year in school, and injury status-including how and when the athlete was injured and if the injury was related to changes in training schedule due to COVID-19-were also collected.

Data collection

Athletes meeting inclusion criteria were contacted before the beginning of their in-season training (e.g., survey links were distributed to the Women’s Soccer team before the Fall 2020 competition season). Emails were sent from the Northwestern University Athletics Department to 13 different athletic teams at Northwestern. Each email presented the study and its objectives and contained a hyperlink to the consent form and the RTS survey. Student-athletes were able to respond directly to the email for additional details. This email also contained instructions to opt out of the study for student-athletes not interested in participating. Reminder emails were sent three weeks following the first email to student-athletes who had not already opted out. All survey data were collected via the RedCap online survey software. This study received IRB approval from the Northwestern IRB Department.

Analysis

The mean, median, range, interquartile range, and frequency distribution were calculated using MS Excel (Redmond, USA). All tables and figures, including the Student’s t-tests for non-parametric samples, were also created using MS Excel.

The primary variables of interest were those pertaining to confidence in returning to sport and the degree to which the COVID-19 pandemic affected aspects of student-athlete life, including mental health, disruption of pre-season training, and access to injury rehabilitation resources. Student’s t and one-way ANOVA tests were performed to compare athletes’ return to sport readiness scores.

## Results

Characteristics of the sample

The characteristics of the sample are summarized in Table 1. Among the 68 participants in the study, the average age was 20.1 years, with a median age of 20 years. Forty-four (64.71%) of the athletes were self-identified females, and the remaining 24 (35.29%) were self-identified males. The vast majority, 52 (76.47%), did not participate in contact sports, with the remaining 16 (23.53%) participating in the contact sports of football or lacrosse. There was a wide spread of athletes from many different sports. Of those with an injury, 14 (82.35%) attributed their injury to changes in their training schedule due to COVID-19 restrictions, and the remaining three (17.65%) did not.

**Table 1:** Frequency of demographic characteristics of Northwestern University Division 1 athletes, January 1, 2020, to January 1, 2021, Northwestern University, Chicago, IL. *Sums may be less than the total due to missing values †Contact sports include Football, Lacrosse, Basketball, Field Hockey, and Soccer ‡Values represent players who have already started or completed their official competition for the 2020-2021 academic year

Patient Characteristics	Total* (N = 68)
Age (in years)	
Mean (Standard Deviation)	20.1 (1.42)
Median [Min, Max]	20 {18, 23}
Gender	
Female	44 (64.71%)
Male	24 (35.29%)
Year in School	
First	15 (22.06%)
Second	18 (26.47%)
Third	15 (22.06%)
Fourth	15 (22.06%)
Fifth	5 (7.35%)
Athletic Status	
New Athletes	19 (27.94%)
Returning Athletes	49 (72.06%)
Contact Sport†	31 (45.59%)
Primary Sport	
Baseball	3 (4.41%)
Basketball	1 (1.47%)
Cross Country	11 (16.18%)
Diving	4 (5.88%)
Fencing	3 (4.41%)
Field Hockey	7 (10.29%)
Football	13 (19.12%)
Golf	4 (5.88%)
Lacrosse	3 (4.41%)
Soccer	7 (10.29%)
Softball	4 (5.88%)
Swimming	5 (7.35%)
Volleyball	3 (4.41%)
*Level of Participation this Season *‡	
Active	39 (57.35%)
Injured Reserve	8 (11.76%)
Red Shirt	5 (7.35%)
Currently recovering from an injury that prevents you from playing?	
Yes	17 (25%)
No	51 (75%)
Do you attribute your injury to changes in your training schedule due to COVID-19 restrictions?	
Yes	14 (20.59%)
No	3 (4.41%)

**Figure 1:**
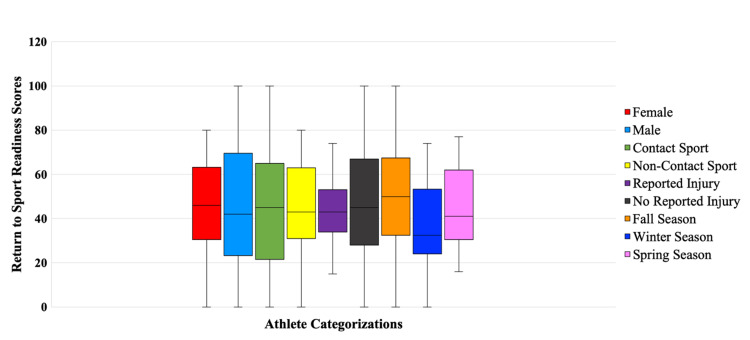
Northwestern University Division 1 return to sport readiness scores by athlete categorization, January 1, 2020, to January 1, 2021, Northwestern University, Chicago, IL.

The results of the readiness to return to sport survey are summarized in Table 2 and Figure 1. Among all athletes, the mean return to sport readiness score was 44 (SD 24.76). The median RTS score was 44. Those playing a winter sport had the lowest mean RTS score, 35 ± 23, and those playing a fall season sport had the highest mean score, 48 ± 25.97. No significant differences in the mean athlete scores were seen between any of the subgroups.

**Table 2:** Northwestern University Division 1 athlete attitudes on COVID-19 impact and readiness to return to sport, January 1, 2020, to January 1, 2021, Northwestern University, Chicago, IL. *Values represent a sum of each player's from the readiness to return to sport survey

	Total N = 68	
	Mean ± SD	p-value
Return to Sport Readiness Score*		
Overall	44 ± 24.76	---
Female	45 ± 22.47	0.754
Male	43 ± 29.33	---
Contact Sport	44 ± 28.15	1.000
Non-Contact Sport	44 ± 22.21	---
Reported Injury	43 ± 16	0.777
No Reported Injury	45 ± 27.39	---
Fall Season Sport	48 ± 25.97	0.204
Winter Season Sport	35 ± 23	---
Spring Season Sport	46 ± 21	---

## Discussion

Overall, competitive athletes on leave from their sport due to collegiate and Division 1 COVID-19 guidelines had lower reported mean RTS scores than athletes outlined in many ACL-RSI score studies. According to the original ACL-RSI validation study outlined by Webster et al., participants who eventually returned to their sport scored an average of 70 points on the ACL-RSI survey, whereas those who did not return scored an average of 46 points [12]. Another ACL-RSI validation study further distinguished between the scores of athletes who returned to competitive sports (71.1 ± 8.9) as opposed to recreational sports (62.9 ± 10.5) [13]. In comparison, our competitive athletes scored, on average, closer to those who did not return to the sport than those who did. Though we did not conduct a follow-up study to see which athletes went on to return to sport or not, we assume that most returned to their season of training and competing once health and regulations allowed. Of course, other factors among our population, such as degree of athleticism, collegiate scholarships, and team coherence, are integral and may have impacted athlete return post-COVID-19. A follow-up study on which of these athletes returned or abstained from their sport and the factors contributing to that decision may be insightful.

Many other ACL-RSI utilization and validation studies report higher average readiness scores than those reported by our athletes, who scored an average of 44 ± 24.76. One such study in Japan drew from a similarly varied spread of athletes from many different sports, reporting an average score of 61.7 ± 18.2 [14]. Similarly, Sala-Barat et al., who worked to translate and cross-culturally adapt the ACL-RSI for Spanish-speaking patients, reported an average score of 64.8 ± 19.5 [15]. The patients in that study were all semi-professional soccer players with similar levels of athleticism to the Division 1 athletes in our study. The study, conducted before the onset of COVID-19, indicates moderately good levels of readiness to return to sport. Though each of these assessments is a slightly different adaptation of the ACL-RSI, our study may uniquely highlight COVID-19 as a more-severe detriment to return to sport readiness than the injury alone, as athletes with self-reported injuries in our study scored an average of 21.8 points lower than the soccer players in this study (p< 0.0001).

One study by Martin et al. examined the differences in ACL-RSI scores in patients who abstained from their sports before and during the COVID-19 pandemic. Interestingly, they found no significant difference in reported readiness to return to sports post-surgery between the two groups but attributed that to the implementation of comprehensive online physical therapy and training programs [16]. The impact of COVID-19 on our athletes may have been different in that most of these participants were forced to abstain from their sport due to a public health emergency rather than injury or a physical inability to play. For this reason, a comprehensive online physical therapy program may not have increased our athletes’ confidence in their ability to return to play. Among our athletes, those with a reported injury did score an average of two points lower than those without an injury; however, this difference was not statistically significant. A study by Sandler et al. found decreased rates of team-sport-related injuries during pandemic closures [17]. However, among the 17 athletes in our study with self-reported injuries, 14 (82.4%) attributed these injuries to changes in their training schedule due to COVID-19 closures. Future studies may address this discrepancy and elucidate rates of pre-closure and post-closure injuries, specifically among these Division 1 athletes.

Our analysis should be interpreted with some limitations. Most notably, our adapted survey, though inspired by the widely validated RTS survey, was not itself validated and has a different number of questions (10 as opposed to 12 in the ACL-RSI) and a different scoring method than the ACL-RSI. The original ACL-RSI is formatted so that each of the 12 questions is on a scale of 0-100, and the final score is the mean of the 12 scores. The primary limitation of our scoring method is that it is less sensitive than the original ACL-RSI survey, only allowing choices from 0-10 as opposed to 0-100; the final score being a sum of each score as opposed to an average of each may introduce additional statistical variability. As well, differences in the culture and society surrounding the athletes in each study should not be overlooked. Such differences between our surveyed athletes and athletes in different countries or within different contexts may influence the severity of reported scores, but this is less likely given the high rates of validity among ACL-RSI scores in different countries. Lastly, our survey was a single-center study and is thus less generalizable to athletes across the United States than studies performed across several institutions.

Looking forward, several questions regarding a return to sport remain. Among those is the impact of pre-pandemic anxiety and depression levels on athletes’ confidence to return to sport in the setting of COVID-19 closures. The early identification and risk stratification of athletes facing psychological barriers to returning to their sport could allow for increased support and counseling, perhaps through peer mentoring groups or rehabilitation. To address this, it is also important to determine the role that telemedicine, alongside evaluations such as the RTS survey, may play in gauging athlete readiness and confidence to return to sport going forward.

## Conclusions

Overall, the athletes surveyed in our study reported much lower levels of readiness to return to sport in the context of COVID than athletes surveyed in other studies, exhibiting the impact of COVID-19 on athletes’ confidence to return to their scheduled sport season. These differences may highlight COVID as a more severe detriment to returning to sport readiness among division-one athletes than recovering from an injury alone. Given such an impact, more research is needed to elucidate the percentage of these athletes that returned to or abstained from their sport, as well as any motivating, facilitating, or detrimental factors in their choice.

## Appendices

Appendix A

**Table 3:** Readiness to Return to Sport (RTS) Survey

Rate the following on a scale from 1 to 10, with 1 being “least confident” and 10 being “most confident”.		
Q.1	Overall confidence to play this season	Required 1 2 3 4 5 6 7 8 9 10 0 Does not apply to me
Q. 2	Confidence to play without pain this season	Required 1 2 3 4 5 6 7 8 9 10 0 Does not apply to me
Q. 3	Confidence to give 100% of my efforts this season	Required 1 2 3 4 5 6 7 8 9 10 0 Does not apply to me
Q. 4	Confidence in my own skills or ability level this season	Required 1 2 3 4 5 6 7 8 9 10 0 Does not apply to me
Q. 5	Confidence to play at the same level as last season	Required 1 2 3 4 5 6 7 8 9 10 0 Does not apply to me
Q. 6	Confidence in my teammates to rely on me to play my best this season	Required 1 2 3 4 5 6 7 8 9 10 0 Does not apply to me
Q. 7	Concern for what others might think if I fail to perform at last year’s skill level during this season	Required 1 2 3 4 5 6 7 8 9 10 0 Does not apply to me
Q. 8	Concern that COVID-19 has jeopardized my future in this sport	Required 1 2 3 4 5 6 7 8 9 10 0 Does not apply to me
Q. 9	Confidence that my injured body part will be able to handle the demands of the sport this season	Required 1 2 3 4 5 6 7 8 9 10 0 Does not apply to me
Q. 10	Concern for exacerbation of a current injury or reinjury	Required 1 2 3 4 5 6 7 8 9 10 0 Does not apply to me
